# Multidrug Resistant *Acinetobacter baumannii*: Risk Factors for Appearance of Imipenem Resistant Strains on Patients Formerly with Susceptible Strains

**DOI:** 10.1371/journal.pone.0009947

**Published:** 2010-04-01

**Authors:** Jung-Jr Ye, Ching-Tai Huang, Shian-Sen Shie, Po-Yen Huang, Lin-Hui Su, Cheng-Hsun Chiu, Hsieh-Shong Leu, Ping-Cherng Chiang

**Affiliations:** 1 Division of Infectious Diseases, Department of Internal Medicine, Chang Gung Memorial Hospital, Taipei, Taoyuan, Taiwan; 2 Division of Clinical Pathology, Department of Pathology, Chang Gung Memorial Hospital Linkou Medical Center, Taipei, Taoyuan, Taiwan; 3 Division of Pediatric Infectious Disease, Department of Pediatrics, Chang Gung Children's Hospital, Taipei, Taoyuan, Taiwan; Charité-Universitätsmedizin Berlin, Germany

## Abstract

**Background:**

Multidrug resistant *Acinetobacter baumannii* (MDRAB) is an important nosocomial pathogen usually susceptible to carbapenems; however, growing number of imipenem resistant MDRAB (IR-MDRAB) poses further clinical challenge. The study was designed to identify the risk factors for appearance of IR-MDRAB on patients formerly with imipenem susceptible MDRAB (IS-MDRAB) and the impact on clinical outcomes.

**Methodology/Principal Findings:**

A retrospective case control study was carried out for 209 consecutive episodes of IS-MDRAB infection or colonization from August 2001 to March 2005. Forty-nine (23.4%) episodes with succeeding clinical isolates of IR-MDRAB were defined as the cases and 160 (76.6%) with all subsequent clinical isolates of IS-MDRAB were defined as the controls. Quantified antimicrobial selective pressure, “time at risk”, severity of illness, comorbidity, and demographic data were incorporated for multivariate analysis, which revealed imipenem or meropenem as the only significant independent risk factor for the appearance of IR-MDRAB (adjusted OR, 1.18; 95% CI, 1.09 to 1.27). With selected cases and controls matched to exclude exogenous source of IR-MDRAB, multivariate analysis still identified carbapenem as the only independent risk factor (adjusted OR, 1.48; 95% CI, 1.14 to 1.92). Case patients had a higher crude mortality rate compared to control patients (57.1% vs. 31.3%, *p* = 0.001), and the mortality of case patients was associated with shorter duration of “time at risk”, i.e., faster appearance of IR-MDRAB (adjusted OR, 0.9; 95% CI, 0.83 to 0.98).

**Conclusions/Significance:**

Judicious use of carbapenem with deployment of antibiotics stewardship measures is critical for reducing IR-MDRAB and the associated unfavorable outcome.

## Introduction

*Acinetobacter baumannii* is an increasingly important nosocomial pathogen with resistance to multiple antimicrobial agents [1], [2]. Multidrug resistant *A. baumannii* (MDRAB) usually retained *in vitro* susceptibility to carbapenems [3], [4]; however, emergence of imipenem resistant MDRAB (IR-MDRAB) occurred since early 90's, which imposed a grave concern in clinical practice as IR-MDRAB was susceptible to few drugs *in vitro* [5]–[9].

There have been many studies on risk factors for the emergence of MDRAB [2], [8]–[15]. Patients with carbapenem resistant strains were compared to those with susceptible ones [9]–[11], [14]. The role of carbapenem might be exaggerated with this straightforward comparison [16], [17]. Other studies took hospitalized patient cohorts as controls and risk factors identified were usually those for nosocomial infection [15]. In addition, antimicrobial selective pressure has never been comprehensively quantified and “time at risk”, the period of time at risk for emergence of resistant strains, has seldom been soundly adjusted. Severity of illness or comorbidities were not included for analysis in some studies [11], [13].

This case control study was designed to identify the risk factors for the common situation of appearance of IR-MDRAB on patients formerly with imipenem susceptible MDRAB (IS-MDRAB). We compared two groups of patients, both with IS-MDRAB at first. IR-MDRAB appeared later in one group but not in the other. Time at risk, quantified antimicrobial selective pressure, severity of illness, comorbidity and demographic data, were incorporated into multivariate analysis. The impact of appearance of IR-MDRAB on clinical outcomes was evaluated as well. The study was deliberately designed for optimization of several important methodological principles of case control studies for risk factor analysis of antibiotic resistance, including control group selection and adjusting for confoundings such as time at risk, quantified antimicrobial selective pressure, severity of illness and comorbidity [16].

## Methods

### Ethics statement

The study was approved by the ethics review board of the Chang Gung Memorial Hospital Linkou Medical Center in September 2008. Patient consent was not obtained because data were analyzed anonymously.

### Cases and controls

Chang Gung Memorial Hospital Linkou Medical Center is a 3000-bed university-affiliated medical center with 308 ICU beds. Identification of *A. baumannii* was by conventional biochemical tests [18]. Susceptibilities to all antimicrobial agents were determined and interpreted according to criteria of the Clinical and Laboratory Standards Institute (CLSI) by disk diffusion susceptibility [19]. Database of microbiology laboratory between August 2001 and March 2005 was reviewed for clinical isolates of MDRAB. Multidrug resistance was defined as resistance to amikacin, gentamicin, piperacillin, cefepime, ceftazidime, aztreonam, and ciprofloxacin. Intermediate susceptibility was considered as resistance.

Hospitalized patients with one culture of IS-MDRAB and subsequent cultures of MDRAB from the same body site, sampled during the same hospitalization and at least 7 days apart, were included. Patients with succeeding clinical isolates of IR-MDRAB were defined as the cases. Those with all subsequent clinical isolates of IS-MDRAB were defined as the controls. Two hospitalizations separated by less than 30 days were taken as one hospitalization. Blood was considered as the same body site of any culture site, since it was possibly the invasive complication of any infected site. Pleural effusion was taken as part of the respiratory tract. Patients may be included for more than 1 time if they met the inclusion criteria during different occasions, such as different admissions or different episodes of the same admission.

### Time at risk

The “time at risk” was defined as the time interval between detection of first IS-MDRAB and detection of first IR-MDRAB for case patients, and the time interval between detection of first and last IS-MDRAB for control patients. It is the period of time at risk for appearance of imipenem resistant strains for each individual patient.

### Demographic data and comorbidities

Age, sex, site of *A. baumannii* growth and comorbidities were gathered by reviewing the medical records. Comorbidities included hepatic dysfunction of a bilirubin concentration over 2.5 mg/dl or liver cirrhosis, renal insufficiency of a creatinine level above 2.0 mg/dl or requirement of dialysis, chronic pulmonary disease, cardiac disease, cerebral vascular accident, diabetes mellitus, immune compromise, hematological or solid organ malignancy, and surgery. Immune compromise was defined as corticosteroid use during the hospitalization (prednisone or equivalent over 20 mg per day for at least 2 weeks), human immunodeficiency virus (HIV) infection or acquired immune deficiency syndrome (AIDS), neutropenia (neutrophil count less than 500 cells/mm3) during the time at risk, use of immunosuppressive agents, including chemotherapeutics, within 30 days prior to *A. baumannii* growth, and concurrent hematological malignancy. Surgical procedures were those within 30 days prior to *A. baumannii* growth.

### Clinical conditions and treatments

Length of ICU stay, ventilator dependence and doses and duration of all antibacterial agents, during the period of the “time at risk”, were documented. Severity of illness was quantified by a modified APACHE (Acute Physiology And Chronic Health Evaluation) II score, recorded within 48 hours before or after the day of first clinical isolate of IS-MDRAB [20]. Data for APACHE II score were not all available for patients of minor disease severity and then the missing data were ignored.

### Statistical methods

All statistical analyses were performed using SPSS version 15.0. Tests performed in univariate analysis were chi-square test or Fisher exact tests for categorical variables and Student *t* test or Mann–Whitney test for continuous variables as appropriate. The Kolmogorov-Smirnov test or Shapiro-Wilk test was used to assess normality as appropriate. Odds ratios (ORs) and 95% confidence intervals (CIs) were calculated. All variables with a *p* value of <0.05 in univariate analysis were included in a logistic regression model for multivariate analysis. All tests were two-tailed, and a *p* value of <0.05 was considered significant in multivariate analysis.

## Results

### Risk factors for appearance of IR-MDRAB

#### *Case control study*

With review of the microbiology laboratory database, we identified in 206 patients 209 episodes of infection or colonization of IS-MDRAB, which were with subsequent cultures of MDRAB. Appearance of IR-MDRAB occurred in 49 (23.4%) episodes but not in the other 160 (76.6%). The former were defined as the cases and the later were defined as the controls.

For the 209 episodes, the mean age was 62±20 year old, presenting a population of seniority. Male predominated (66%) and comorbidities were common. About 40% of them received surgical procedures within 30 days prior to *A. baumannii* growth. The “time at risk” ranged from 7 to 134 days, with a mean of 21.9 days. ICU admissions during the “time at risk” were common (60.8%), as well as the use of mechanical ventilation (47.8%). Disease severity varied with a mean APACHE II score of 18.3. Respiratory tract is the predominant site of growth (57.9%). Secondary bacteremia occurred in 16 (7.8%) of the 205 episodes of defined primary site other than primary bacteremia. In-hospital and 30-day mortality rates were 37.3% and 33% respectively.

Comparing cases with controls, there was no difference in predominant sex, but the cases were older (median [quartiles]: 73 [59; 79] vs. 65 [44.3; 77] years, *p* = 0.02). More case patients were with isolates from the respiratory tract (87.8% vs. 48.8%, *p*<0.001) and more control patients were with isolates from the wound (45.0% vs. 8.2%, *p*<0.001). Incidence of bacteremia was comparable for cases and controls (12.2% vs. 8.8%, *p*>0.05). The comorbidities were similar, except that there were more cases with hepatic dysfunction (24.5% vs. 11.3%, *p* = 0.02) and more controls with musculoskeletal system and soft tissue surgeries (30.0% vs. 8.2%, *p* = 0.002). Case patients were with longer time at risk (20 [12; 32.5] vs. 14 [9.3; 24] days, *p* = 0.02), greater ICU length of stay (11 [4.5; 19] vs. 2.5 [0; 10] days, *p*<0.001), more ventilator days (10 [0; 16] vs. 0 [0; 8] days, *p*<0.001), and higher APACHE II scores (24 [18; 31] vs. 17 [7]; [24], *p*<0.001).

The use of antimicrobial agents was reviewed, with doses and days of use precisely verified. Case patients were exposed to more vancomycin or teicoplanin (7 [0.5; 11] vs. 0 [0; 7.8] days, *p*<0.001), imipenem or meropenem (10 [6]; [15] vs. 0 [0; 6] days, *p*<0.001), and clindamycin (0 [0; 2] vs. 0 [0; 0] days, *p* = 0.01). In contrary, control patients were exposed to more first generation cephalosporins (0 [0; 3] vs. 0 [0; 0] days, *p* = 0.004) and gentamicin (0 [0; 5] vs. 0 [0; 0] days, *p* = 0.001) (Table 1). Among all the risk factors identified in univariate analysis, exposure to carbapenems, either imipenem or meropenem, was the only significant independent one for the appearance of IR-MDRAB with multivariate analysis (adjusted OR, 1.18; 95% CI, 1.09 to 1.27) (Table 1).

**Table 1:** Univariate and multivariate analyses of risk factors for appearance of IR-MDRAB.

Variables	Cases (n = 49)^a^	Controls (n = 160)^a^	Univariate	Multivariate^b^	
			*p-*Value	*p-*Value	Adjusted OR (95% CI)
**Demographic parameters**					
Age, years	73 [59; 79]	65 [44.3; 77]	0.02	0.87	1.00 (0.97–1.03)
Male gender	35(71.4)	103(64.4)	0.36		
Site of isolates^c^					
Respiratory tract	43(87.8)	78(48.8)	<0.001	0.37	2.36 (0.36–15.55)
Wound	4(8.2)	72(45.0)	<0.001	0.19	0.20 (0.02–2.17)
Bacteremia	6(12.2)	14(8.8)	0.58		
**Concomitant diseases**					
Hepatic dysfunction	12(24.5)	18(11.3)	0.02	0.30	1.78 (0.60–5.27)
Renal insufficiency	17(34.7)	48(30.0)	0.54		
Chronic pulmonary disease	12(24.5)	28(17.5)	0.28		
Cardiac disease	8(16.3)	17(10.6)	0.28		
Cerebral vascular accident	19(38.8)	56(35.0)	0.63		
Diabetes mellitus	15(30.6)	53(33.1)	0.74		
Immune compromise	11(22.4)	25(15.6)	0.27		
Malignancy					
Hematological malignancy	0(0)	1(0.6)	1.0		
Solid tumor with metastasis	3(6.1)	3(1.9)	0.14		
Solid tumor, no metastasis	5(10.2)	5(3.1)	0.06		
Surgery					
Musculoskeletal/Soft tissue	4(8.2)	48(30)	0.002	0.67	1.43 (0.27–7.50)
Vital organs	8(16.3)	25(15.6)	0.91		
**Clinical conditions in TAR**					
Duration of TAR, days	20 [12; 32.5]	14 [9.3; 24]	0.02	0.77	1.00 (0.96–1.03)
ICU stay, days	11 [4.5; 19]	2.5 [0; 10]	<0.001	0.56	1.03 (0.94–1.13)
Ventilator use, days	10 [0; 16]	0 [0; 8]	<0.001	0.32	0.95 (0.87–1.05)
APACH II Score	24 [18; 31]	17 [7]; [24]	<0.001	0.43	1.02 (0.97–1.08)
**Antibiotic exposure, days^c^**					
Teicoplanin/Vancomycin	7 [0.5; 11]	0 [0; 7.8]	<0.001	0.70	0.99 (0.93–1.05)
1^st^ generation cephalosporins	0 [0; 0]	0 [0; 3]	0.004	0.27	0.86 (0.66–1.12)
Ceftriaxone	0 [0; 2]	0 [0; 0]	0.49		
Imipenem/Meropenem	10 [6]; [15]	0 [0; 6]	<0.001	<0.001	1.18 (1.09–1.27)
Gentamicine	0 [0; 0]	0 [0; 5]	0.001	0.76	1.03 (0.86–1.22)
Metronidazole	0 [0; 0]	0 [0; 2]	0.21		
Clindamycin	0 [0; 2]	0 [0; 0]	0.01	0.68	1.04 (0.87–1.24)
Ciprofloxacin	0 [0; 3.5]	0 [0; 1.8]	0.15		

NOTE. ^a^Categorical data are no.(%) of subject, continuous data are expressed as mean (SD) or median [quartiles].

b All variables included in the final multivariable model are shown.

c Only significant (*p*<0.05) and selected non-significant variables in univariate analysis are shown.

OR  =  odds ratio; CI  =  confidence interval; TAR  =  time at risk; ICU  =  intensive care unit; APACHE II  =  Acute Physiology and Chronic Health Evaluation II; SD  =  standard deviation.

#### *Matched case control study*

In order to exclude the potentially confounding exogenous source of IR-MDRAB, 20 cases and 20 controls were selected from above for a matched case control study. Selected cases and controls were matched for age, location at the hospital, and date of hospitalization, presumably with the same likelihood of acquiring exogenous IR-MDRAB. Ages were matched for either below or above 55 and locations and date of hospitalization were matched for admission to the same ICU within 45 days. For these cases and controls, sites of isolates were comparable, with respiratory tract predominated for both groups (95% vs. 85%, *p*>0.05). Gender, comorbidities, ICU length of stay, ventilator days, and APACHE II scores were also similar. “Time at risk” was longer for cases than controls (21.5 [12.3; 37.3] vs. 10.5 [9.3; 19.3] days, *p* = 0.02). Vancomycin or teicoplanin (7.5 [1]; [17] vs. 0 [0; 7.8] days, *p* = 0.03), imipenem or meropenem (10 [8]; [16] vs. 0 [0; 7.8] days, *p*<0.001) and ciprofloxacin (2 [0; 5] vs. 0 [0; 1.5] days, *p* = 0.03) were more often used in case patients. Multivariate analysis reached the same conclusion that exposure to carbapenems was the only significant independent risk factor for the appearance of IR-MDRAB (adjusted OR, 1.48; 95% CI, 1.14 to 1.92) (Table 2).

**Table 2:** Matched univariate and multivariate analyses of risk factors for appearance of IR-MDRAB.

Variables	Cases (n = 20)^a^	Controls (n = 20)^a^	Univariate	Multivariate^b^	
			*p-*Value	*p-*Value	Adjusted OR (95% CI)
**Demographic parameters**					
Male gender	12(60)	14(70)	0.51		
Site of isolates^c^					
Respiratory tract	19(95)	17(85)	0.61		
Wound	1(5)	1(5)	1.0		
Bacteremia	2(10)	1(5)	1.0		
**Concomitant disease^c^**					
Renal insufficiency	7(35)	5(25)	0.49		
Chronic lung disease	5(25)	8(40)	0.31		
Diabetes mellitus	7(35)	13(65)	0.06		
Immune compromise	6(30)	5(25)	0.72		
Solid tumor	3(15)	1(5)	0.61		
**Clinical conditions in TAR**					
Duration of TAR, days	21.5 [12.3; 37.3]	10.5 [9.3; 19.3]	0.02	0.27	0.96 (0.90–1.03)
ICU stay, days	12.5 [7.5; 21.8]	10 [8.3; 16]	0.43		
Ventilator use, days	11.5 [6; 20.3]	8 [2.3; 14]	0.18		
APACH II Score	26.80 (8.7)	24.65 (8.0)	0.42		
**Antibiotic exposure, days^c^**					
Teicoplanin/Vancomycin	7.5 [1]; [17]	0 [0; 7.8]	0.03	0.08	0.87 (0.75–1.01)
Ceftriaxone	0 [0; 0]	0 [0; 5]	0.08		
Imipenem/Meropenem	10 [8]; [16]	0 [0; 7.8]	<0.001	0.003	1.48 (1.14–1.92)
Clindamycin	0 [0; 2]	0 [0; 1.5]	0.44		
Ciprofloxacin	2 [0; 5]	0 [0; 1.5]	0.03	0.70	1.05 (0.81–1.37)

NOTE. ^a^ Categorical data are no.(%) of subject, continuous data are expressed as mean (SD) or median [quartiles].

b All variables included in the final multivariable model are shown.

c Only significant (*p*<0.05) and selected non-significant variables in univariate analysis are shown.

OR  =  odds ratio; CI  =  confidence interval; TAR  =  time at risk; ICU  =  intensive care unit; APACHE II  =  Acute Physiology and Chronic Health Evaluation II; SD  =  standard deviation.

### Clinical outcomes

#### *Mortality*

The case patients had higher in-hospital and 30-day mortality (57.1% vs. 31.3%, *p* = 0.001 and 49.0% vs. 28.1%, *p* = 0.007). Case patients who survived were more likely with isolates from the wound (19% vs. 0%, *p* = 0.03). MDRAB bacteremia was noted in 6 of the 49 cases, with 5 of them of IR-MDRAB. In-hospital mortality for the 5 patients was 80% (4/5). One patient of chronic ulcer infection survived, even without specific antimicrobial therapy.

#### *Risk factors for mortality*

Associations between in-hospital mortality and various conditions during the period of “time at risk” were analyzed for the 49 cases with appearance of IR-MDRAB. Cases with mortality had more hepatic dysfunction (39.3% vs. 4.8%, *p* = 0.005), higher APACH II scores (mean±standard deviation, 27.6±7.4 vs. 20±9, *p* = 0.002), longer ventilator days (11 [9; 18.8] vs. 3 [0; 13] days, *p* = 0.02), less exposure to oxacillin (*p* = 0.02), and shorter “time at risk” (15.5 [10; 22.5] vs. 24 [19; 35.5] days, *p* = 0.004). However, in-hospital mortality was significantly associated only with the length of “time at risk” in multivariate analysis (adjusted OR, 0.9; 95% CI, 0.83 to 0.98, logistic regression of backward stepwise procedure). The sooner the IR-MDRAB emerged, the less chance the case patients would survive.

#### *Outcome with specific treatment*

Combination of carbapenem and sulbactam is one of the recommended regimens for IR-MDRAB eradication, based on the *in vitro* synergism and the *in vivo* effectiveness in an animal model [21], [22]. Forty two case patients who survived for more than 3 days after emergence of IR-MDRAB were included for analysis of treatment outcome with specific regimens. Patients who received the combination of carbapenem and sulbactam for at least 3 days had higher in-hospital mortality but this was with no statistical significance (6/8 75% vs. 16/34 47.1%, *p* = 0.24).

## Discussion

Our study reached the conclusion that the only independent risk factor for appearance of IR-MDRAB in patients formerly with IS-MDRAB is the use of carbapenem. The “time at risk”, the period of time at risk for appearance of IR-MDRAB, is a very important confounding factor to be adjusted because the probability of appearance increases with the length of the time [16]. In our study the “time at risk” was precisely defined for each individual patient and analyzed. “Time at risk” is also the critical period of time when physicians have to make decision on whether to eradicate IS-MDRAB with carbapenems. Antimicrobial selective pressure and clinical parameters, such as APACHE II score, ICU stay, and ventilator use were focused on the period of “time at risk”. Although old age, respiratory tract isolates, prolonged ICU stay, prolonged ventilator use and high APACHE II score were all significant risk factors in univariate analysis, multivariate analysis revealed carbapenem only as the significant independent risk factor. Some APACHE II score data was not complete, particularly for those not staying in the ICU. Low scores may have been underestimated and then the high scores of critically ill patients were relatively over-estimated. Even with over-estimation, APACHE II score still could not stand out as an independent risk factor in the multivariate analysis.

The clinical impact of MDRAB is controversial. Some studies showed prolonged hospital and ICU stays and increased mortality; however, there was no attributable mortality if severity of illness and underlying diseases had been controlled [12], [23]. Our case patients had higher mortality in univariate analysis and the length of “time at risk” was the only significant independent risk factor for mortality in multivariate analysis. That is, patients with IR-MDRAB were more likely to incur mortality if IR-MDRAB had appeared in a faster pace. This result implied unfavorable outcome with appearance of IR-MDRAB.

Our samples were clinical, instead of surveillance, isolates. All cultures were requested by clinicians based on clinical discretion. Studies with surveillance isolates may reach different conclusions. However, clinical isolates are more clinically relevant and will provide insightful information for clinical practice. The serial isolates were from the same body sites of each individual patient. It implied possibility of either susceptibility conversion from formerly imipenem susceptible strains to resistance or resistant strain selection. It was also possible that some resistant strains were acquired from an exogenous source. Our effort for this problem was to select cases and controls for a matched control study. The selected cases and controls, matched for age and location and date of hospitalization, were with similar theoretical likelihood of acquiring resistant strain from the environment. Besides, IR-MDRAB on subsequent cultures of less than 7 days were excluded by our arbitrary definition that they were more likely from exogenous sources.

It is interesting to understand the microbiology behind this clinical phenomenon. *Acinetobacter* species possessed a wide array of β-lactamases and other enzymes for carbapenem resistance, encoded by transposable elements, plasmids or chromosomes [24]. One possibility was that antimicrobial selective pressure caused conversion of the sensitive clones, either by mutation or by induced expression of resistance elements which had been kept silent otherwise. It is also possible that carbapenem-resistant *Acinetobacter* expanded from undetectable minority to predominant clones as carbapenem had eradicated the majority of sensitive ones. Although our data somehow suggested an inducible mechanism for carbapenem resistant strains to emerge under the selective pressure of carbapenem, there has been so far no microbiological evidence linking any resistance mechanism to such a phenomenon.

Numerous reports suggested causal associations between quantities of antibiotic used and the development of resistance with hospital-wide or nationwide data [25], [26]. The direct relationship between antibiotic consumption and emergence of resistance on individual patient was not clear. Our conclusion that carbapenem use was the only independent risk factor for appearance of IR-MDRAB exemplified a dilemma in clinical practice. We have to use carbapenem for eradication of IS-MDRAB and it actually causes the emergence of IR-MDRAB at the same time. Other antimicrobial agents should be evaluated for the efficacy of eradicating IS-MDRAB. Effective alternative therapy would decrease emergence of IR-MDRAB. It can not be overemphasized that the decision to eradicate IS-MDRAB with carbapenem has to be exercised with caution. Judicious use of carbapenem with antibiotics stewardship programs would be the most effective measure to avoid the emergence of imipenem resistant MDRAB and the associated unfavorable outcome.
